# Does capsular repair make a difference in the integrity and thickness of anterior capsule in the setting of borderline hip dysplasia

**DOI:** 10.1186/s12891-023-06307-y

**Published:** 2023-03-13

**Authors:** Fan Yang, Hong-Jie Huang, Xin Zhang, Jian-Quan Wang

**Affiliations:** Department of Sports Medicine, Beijing Key Laboratory of Sports Injuries, Peking University Third Hospital, Institute of Sports Medicine of Peking University, Haidian District, 49 North Garden Rd, 100191 Beijing, P. R. China

**Keywords:** Borderline developmental dysplasia of the hip, Anterior capsular defect, Anterior capsular thickness, Magnetic resonance imaging

## Abstract

**Background:**

Hip joint capsular ligaments serve a fundamental role in balancing functional mobility and joint stability. However, few studies had focused on postoperative capsule changes in patients with borderline developmental dysplasia of the hip (BDDH).

**Purpose:**

To evaluate the integrity and thickness of anterior hip capsular thickness on pre and postoperative MRI in BDDH patients.

**Study design:**

Case series study; Level of evidence III.

**Methods:**

A retrospective analysis was performed using data from BDDH patients who had arthroscopy between 2016 and 2019. Two groups were created and propensity-score matched based on whether the capsule was sutured. The study group comprised patients who have undergone routine capsule repair between 2018 and 2019. The control group includes BDDH patients with unrepaired capsulotomy between 2016 and 2018. Capsular integrity and thickness were measured on MRI before surgery and at least one year postoperatively. Furthermore, analysis was performed on correlations between the presence of a capsular defect and related factors.

**Results:**

Propensity-score matching yielded 37 hips in the repair group and 37 hips in the non-repair group. There were no significant differences detected in age, sex, and BMI between the two groups. MRI detected capsular defects in 3 hips (8.1%) in the repair group and 10 hips (27.0%) in the non-repair group (*p* = 0.032). The defect was found to be along the interportal capsulotomy line in all capsular defect cases. Moreover, the postoperative anterior capsule thickness in the study group was significantly thinner compared with preoperative (2.9 ± 0.5 mm vs 3.7 ± 0.6 mm; *p* < 0.001), and no significant difference was detected in the control group. There were no statistically significant correlations between the presence of a defect capsule and demographic characteristics such as patient age, sex, BMI, preoperative alpha angle, or lateral center–edge angle (LCEA).

**Conclusion:**

The majority (91.9%) of the repaired hip capsules in BDDH patients remained closed compared with patients without repair (73.0%). The anterior capsule was significantly thinner in the zone of capsulotomy postoperative compared with preoperative in patients with unrepaired capsules. The presence of a defective capsule does not correlate with demographic factors.

## Introduction

Hip arthroscopic surgery has become a common procedure for the correction of femoroacetabular impingement syndrome (FAIS). The incidence of hip arthroscopic surgery in FAIS patients increased by 85% between 2011 and 2018 in the United States [1]. Hip arthroscopic surgery and its indications continue to expand recently. Developmental dysplasia of the hip (DDH) is a common musculoskeletal condition and is considered a precursor to osteoarthritis (OA), which is mainly treated by periacetabular osteotomy (PAO). This condition can be further classified based on the extent of acetabular coverage as severe, moderate, mild, or borderline (BDDH) [2]. BDDH is commonly defined as a lateral center–edge angle (LCEA) with a lower threshold of 18° to 22° or 25° [2, 3]. The role of arthroscopy in the treatment of patients with dysplasia remains controversial. There is also a growing trend among hip arthroscopic surgeons to surgically treat patients with BDDH [4]. Previous studies have reported that BDDH patients can also achieve clinically significant outcomes in short-term and midterm follow-up after arthroscopic treatment compared with normal acetabular coverage FAIS patients [4, 5].

Capsular management in the setting of hip arthroscopic surgery had received an increasing amount of attention, with an emerging interest in improving capsular management for hip preservation [6]. Hip joint capsular ligaments serve a fundamental role in balancing functional mobility and joint stability. Iliofemoral ligament is the strongest capsular ligament and is considered to be a primary stabilizer of the hip joint [7]. Routine capsulotomy during hip arthroscopic surgery can disrupt the iliofemoral ligament. Moreover, biomechanical studies have shown that compared to the intact capsule, capsulotomy increases joint mobility, which can be restored to normal joint mobility with capsular repair [8–10]. However, controversy still surrounds the need for capsular repair following this surgical intervention. Strickland et. al reported that unrepaired capsulotomies can heal within 24 weeks postoperatively on magnetic resonance imaging (MRI) [11]. Domb et. al reported that the use of capsular repair did not show clinically relevant superiority over unrepaired capsulotomy [12]. In contrast, a randomized controlled trial showed that patients undergoing complete capsular closure achieved improved PROs compared with both unrepaired T-capsulotomy and interportal capsulotomy [13]. However, few studies have focused on the changes in the integrity and thickness of the capsule after hip arthroscopic surgery on MRI in BDDH patients. This sparked speculation on whether the capsule could heal and be restored to normal thickness after closure during hip arthroscopic surgery in BDDH patients.

The purpose of this study was to evaluate the integrity and thickness of anterior hip capsular on preoperative and postoperative MRI in BDDH patients. The hypothesis was that BDDH patients with routine capsular repair would have a significantly higher proportion of healing and thicker anterior capsules on MRI compared with patients without repair.

## Methods

### Patient selection and imaging

Approval for the study was granted through the hospital review board IRB. We used retrospective data for patients without capsular closure performed at our institution from 2016 to 2018 and counterparts with routine capsular repair between 2018 to 2019. Inclusion criteria included: primary hip arthroscopic surgery with LCEA from 20° to 25°, 16 to 55 years of age, and a postoperative MRI of the operative hip at least one year after surgery. Exclusion criteria included: revision hip surgery, moderate to advanced osteoarthritis (Tӧnnis grade ≥ 2), and incomplete radiographs and medical records.

The patients had undergone preoperative anteroposterior (AP) pelvis and 45° Dunn lateral radiography. Radiographic measurements were performed using a picture archiving and communication system (PACS; GE Healthcare). The alpha angle was measured on 45° Dunn lateral radiography. The LCEA angle was measured on AP pelvis radiographs, with an LCEA angle between 20° and 25° indicating BDDH [5].

### Evaluation of anterior capsular thickness on preoperative and postoperative MRI

**Fig. 1:**
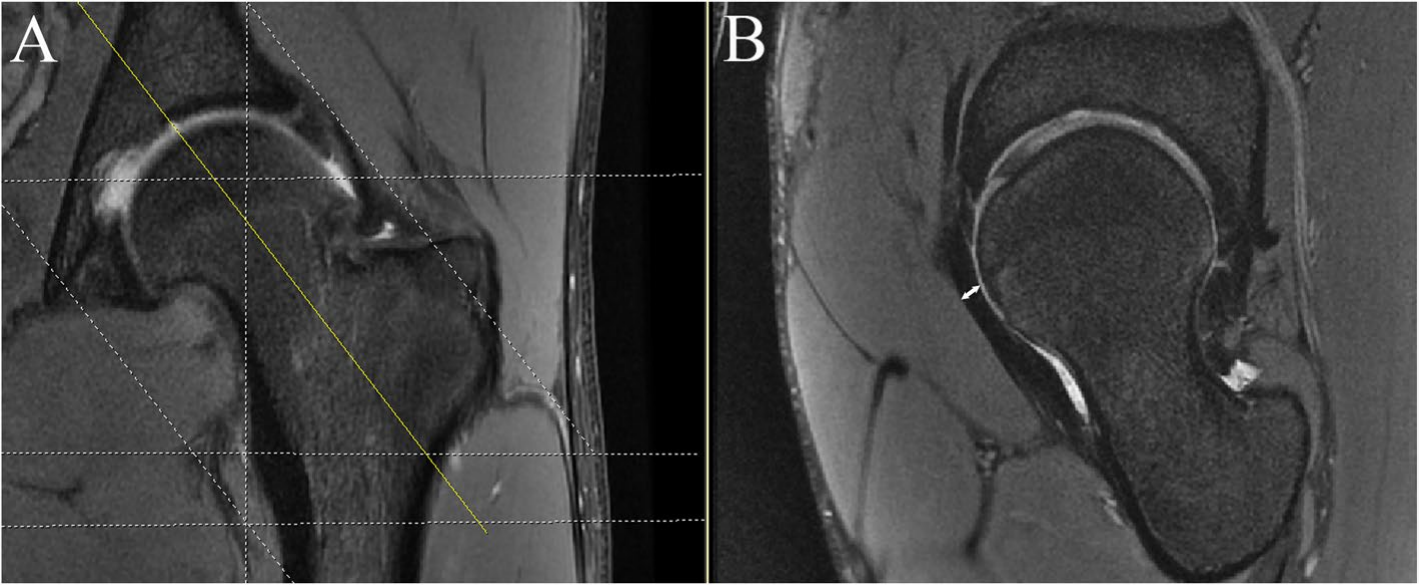
MRI measurement of the capsular thickness. **A**, stereo correlation to locate the oblique-sagittal sequence; **B**, measurement of the anterior capsular thickness (white line)

Preoperative MRI was performed on a 3.0-T system (Magnetom Trio Tim, Siemens) to evaluate the status of labral and articular cartilage. Patients were in the routine supine position. The affected hip joint was surrounded by a soft wrapped surface coil. The scanning scheme consisted of the following sequences: (1) axial oblique views; (2) oblique coronal views (ST: 3.5 mm, TR: 2,505 ms, TE: 37 ms, matrix: 512 × 512). Anterior capsule thickness was measured on proton density-weighted oblique-sagittal sequence through the anterior inferior iliac spine (AIIS) at the site of routine capsulotomy [14]. Stereo correlation localization was used to determine the accurate section on oblique-sagittal sequence views (Fig. 1). A capsular defect was defined as any visual disruption in the iliofemoral ligament in the zone of capsulotomy or the appearance of communication between the joint and the iliopsoas bursa [15].

The patients were divided into two groups based on whether the capsule was sutured or not. A 1:1 propensity-score match based on age, sex, body mass index (BMI), and follow-up time was performed to control for potential confounding variables in the study group and the control group. All MRI images were evaluated by two musculoskeletal radiologists. Both observers were blinded to all clinical data of patients. They took two measurements three weeks apart to ensure the production of reliable and clinically meaningful results.

### Surgical procedures

All procedures were performed by one senior author. The patient was placed in the supine position on standard hip traction (Smith & Nephew, Memphis, TN, USA). The perineum was protected, and the limb undergoing surgery was tractioned with an 8–10 mm distract for hip joint space. The procedure began with fluoroscopic localization of the anterolateral (AL) portal, midanterior portal (MAP), and the proximal mid-anterior portal (PMAP), using a 70° arthroscope. An interportal capsulotomy was performed for all patients in the study cohort. Most pathology in the central compartment, including pincer deformity, labral injury, and chondrolabral injury can be treated. Cartilage damage was assessed using the Outerbridge and acetabular labrum articular disruption (ALAD) classification systems [16]. After addressing pathology in the central compartment, an arthroscope was introduced into the peripheral compartment for decompression of the Cam deformity using a high-speed burr (Smith & Nephew, Andover, Massachusetts). If other extra-articular pathologies are involved, such as ischiofemoral impingement (IFI), gluteus medius tears, and subspine impingement syndrome (SSI), corresponding treatments were also performed. The capsule was without closure before 2018. Since 2018, capsular plication [17] was performed with approximately 3 to 4 interrupted stitches at the end of the procedure.

### Postoperative rehabilitation

All patients followed a well-standardized prescribed rehabilitation protocol under the supervision of the physiotherapy team as previously described. Rehabilitation was divided into phases and took an average of 4 to 5 months. Briefly, the first phase comprised isometric contractions (the ankle pump, quadriceps, and hip joint muscle isometric contraction exercises) and passive range-of-motion exercises. Partial weight-bearing was initiated on the second postoperative day. The second phase focused on maintaining a regular gait and restoring a full range of motion, including adduction, abduction, and pronation. The third phase was about regaining lower extremity strength as well as normal functional activities. The final phase focused on resuming pre-injury higher-level activities.

### Statistical analysis

The Shapiro–Wilk test was used to ensure that all parametric statistical assumptions were satisfied. A 2-tailed unpaired Student t-test was used to compare continuous demographic data between the two groups. Chi-square test or Fisher exact test was used to compare categorical variables between the two groups. The Spearman rank correlations were used to determine the associations between the presence of a defect capsule and related factors. Intraobserver and interobserver reliability were calculated using the intraclass correlation coefficient (ICC), with < 0.40 classified as poor, 0.40–0.59 as fair, 0.60–0.74 as good, and > 0.75 as excellent. SPSS version 26 (IBM, Armonk, NY) was used for statistical analyses. *P*-values less than 0.05 were considered statistically significant. A post-hoc analysis using G*Power (version 3.1) was performed for determining the adequacy of the sample size.

## Results

### Characteristics of the patients

Propensity-score matching yielded 37 hips in the study group and 37 hips without routine capsule repair in the control group. There were no significant differences detected in age, sex, and BMI between the two groups. The demographic and radiographic data of all patients were presented in Table 1. There was 24 female (64.9%) and 13 male (35.1%) patients in both groups. Mean preoperative LCEA was 24.0° and 23.9° for the study and control groups, respectively.

**Table 1:** Characteristics of the patients

Category	Non-repair group	Repair group	*P*-Value
No. of hips	37	37	
Age, yr	37.4 ± 12.1	35.2 ± 12.1	0.441
BMI, kg/m2	22.4 ± 2.7	22.7 ± 4.0	0.641
Sex, n (%)			> 0.999
Female	24 (64.9)	24 (64.9)	
Male	7 (35.1)	7 (35.1)	
MRI follow-up time, m	18.4 ± 7.2	18.7 ± 5.5	0.843
Alpha Preoperative	58.4 ± 5.5	56.4 ± 5.1	0.116
Alpha Postoperative	46.4 ± 5.4	45.1 ± 5.1	0.309
Smoker	2 (5.4)	4 (10.8)	0.670
Cam impingement	37 (100)	37 (100)	> 0.999
LCEA Preoperative	23.1 ± 3.7	23.2 ± 1.6	0.942
Postperative capsular defect, n (%)	10 (27.0)	3 (8.1)	**0.032**
Pre-op anterior capsular thickness(mm)	3.8 ± 0.3	3.9 ± 0.4	0.097
Post-op anterior capsular thickness(mm)	2.9 ± 0.5	3.7 ± 0.6	**< 0.001**

Values are given as mean ± SD

*BMI* Body mass index, *LCEA* Lateral center edge angle

Bold value indicates statistical significance

### Capsular defects

**Fig. 2:**
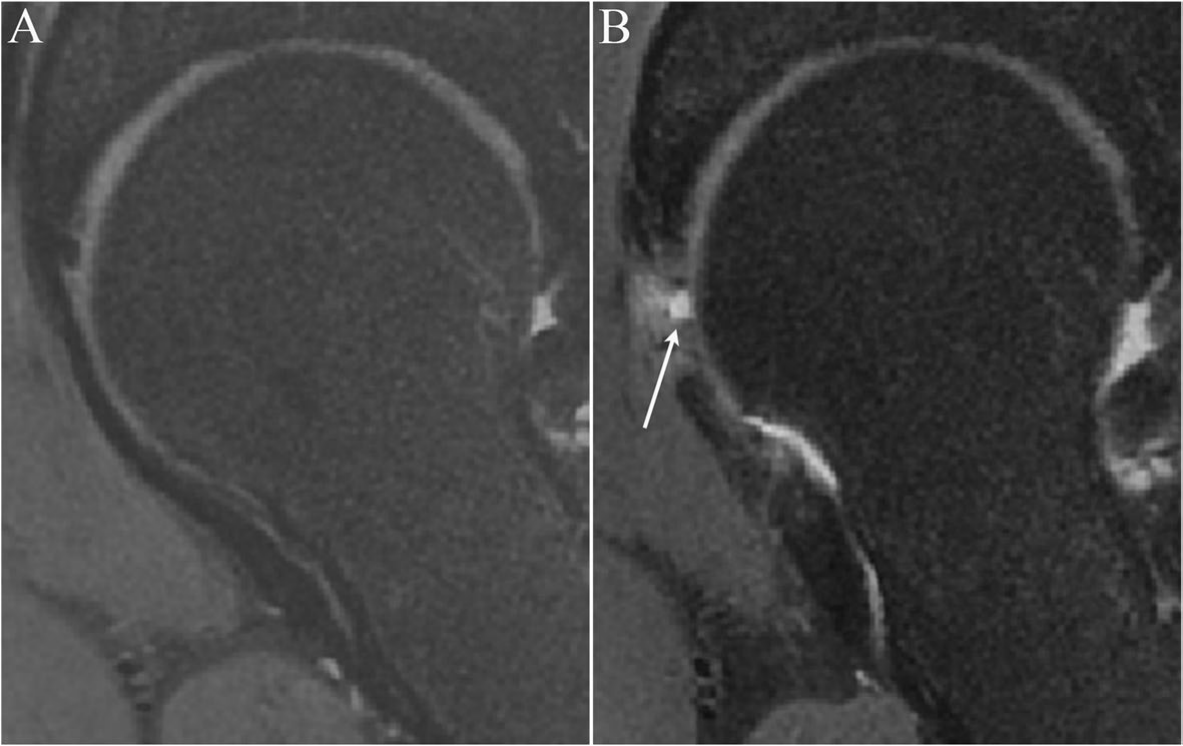
MRI measurement of pre- and postoperative anterior capsular thickness in one same patient. A, preoperative intact capsule; B, postoperative unhealed capsule (white line indicates the site of interportal capsulotomy)

Patients with unrepaired capsules had a higher proportion of unhealed capsules (*p* = 0.032). MRI revealed capsular defects in 3 patients (3/37, 8.1%) with routine capsular closure between 2018 and 2019. Of the 37 patients without routine capsular closure between 2016 and 2018, MRI detected capsular defects in 10 patients (27.0%). The defect was found to be along the interportal capsulotomy line in all capsular defect cases (Fig. 2). There were no statistically significant correlations between the presence of a defect capsule and demographic characteristics such as patient age, sex, BMI, preoperative alpha angle, or LCEA angle (Table 2).

**Table 2:** Correlations between the presence of a defective capsule and related factors

	Capsule defect	
	ρ	*P* Value
Age	0.047	0.694
sex	-0.042	0.721
BMI	-0.027	0.817
MRI follow-up time	-0.082	0.488
Preoperative alpha angle	-0.011	0.927
Preoperative LCEA angle	-0.060	0.611

*BMI* Body Mass Index, *ρ* Spearman rank coefficient

### Anterior capsular thickness

The postoperative anterior capsule thickness in the non-repaired group was significantly thinner in the zone of capsulotomy compared with preoperative (2.9 ± 0.5 mm vs 3.7 ± 0.6 mm; *p* < 0.001). In the repaired group, it was 3.9 ± 0.4 mm and 3.7 ± 0.6 mm before and after operation respectively (*p* = 0.097). The ICC found that the intraobserver and interobserver reliability of capsule thickness was presented in Table 3, indicating a high level of reliability. Moreover, the post hoc power analysis revealed sample size was adequate. Setting alpha to 0.05, using the means (2.9 and 3.7) and standard deviations (0.5 and 0.6) of postoperative capsular thickness, beta (power) was determined to 0.99.

**Table 3:** ICC of capsular thickness measurements

Measurement	Interobserver	Intraobserver
Pre-op capsule thickness	**0.761**	**0.701**
Post-op capsule thickness	**0.932**	**0.944**

*ICC* Intraclass correlation coefficient

## Discussion

The main findings of this study were that arthroscopic repair of interportal hip capsulotomy yields an insignificant increase in the percentage of capsules healing and the Integrity on MRI in BDDH populations. The joint capsule restored its integrity in the majority (91.9%) of BDDH patients receiving capsule repair during hip arthroscopic surgery after 1 year postoperatively, which was superior to the BDDH patients with unrepaired capsules (73.0%). There were no significant correlations found between the presence of a defect capsular and patient demographic characteristics such as patient age, sex, BMI, preoperative alpha angle, or preoperative LCEA.

The capsular ligament can reinforce stability and protect the joint from edge-loading [7]. Partial capsular repair improves hip stability marginally, only a complete repair can restore the hip's native biomechanical profile [18]. Interestingly, Strickland et al. [11] reported that repaired and unrepaired capsulotomy sites progressed to healing with a contiguous appearance on MRI by 24 weeks postoperatively, that differs from our results. However, they excluded patients with hip dysplasia and borderline dysplasia. BDDH is an increasingly recognized factor predisposing to microinstability, and hip microinstability often correlates with a thinning capsule on MRI [19]. After hip arthroscopic surgery, the state of instability may persist and cause repetitive strains on the hip capsule, which might hinder the healing process postoperatively.

It remains controversial whether capsulotomies should be performed during hip preservation surgery, especially when capsular contracture may contribute to pathological processes. Diseased hips may have thicker capsules but dysplasia hips had significantly thinner capsules compared to normal acetabular coverage patients. Rakhra et al. compared the capsular thickness in normal acetabular coverage diseased surgical hips with asymptomatic control hips. They found the capsule is thicker in diseased surgical hips compared with asymptomatic control hips [20]. Hui et al. measured capsular thickness in different groups including dysplasia hips (DDH), borderline dysplasia hips, and FAIS patients. The results show that dysplasia hips have a significantly reduced capsular thickness on MRI, but there was no significant difference in capsular thickness between the BDDH and the FAIS groups. They contributed this to the sequence of instability, they speculated that the thinner capsular thickness could be related to the repetitive strains resulting from hip instability [14]. It is known that borderline hip dysplasia is an increasingly recognized factor predisposing to microinstability. The capsule can provide important restraints to femoral head motion, and iatrogenic defects can predispose patients to instability after surgery [19]. Our results showed that routinely repairing allows a greater likelihood of healing of the capsule among BDDH patients which may lead to better hip stability and clinical outcomes.

### Limitation

This study has certain limitations. First, the retrospective nature of the study introduces innate bias associated with the study design. Though a post hoc power analysis was performed, the sample size is relatively small, and the findings may not be clinically relevant. Second, the time points of MRI follow-up are different, and the integrity and thickness of the capsule may be different at different time points. However, a previous study has shown that the capsule will heal within 6 months [15]. Third, patients reported outcomes were not compared between the two groups. Because the two groups of patients were selected from different periods, there are many potentially confounding factors. Further study is needed to evaluate the effect of postoperative capsular integrity and thickness on clinical outcomes in BDDH patients.

## Conclusion

The majority (91.9%) of the repaired hip capsules in BDDH patients remained closed compared with patients without repair (73.0%). The anterior capsule was significantly thinner in the zone of capsulotomy postoperative compared with preoperative in patients with unrepaired capsules. The presence of a defective capsule does not correlate with demographic factors.

## Data Availability

The datasets used and/or analysed during the current study are available from the corresponding author on reasonable request.

## Abbreviations

DDH: Developmental dysplasia of the hip
BDDH: Borderline developmental dysplastic hip
FAI: Femoraacetabular impingement
